# Study on the Mechanism of Molecular Weight Reduction of Polyethylene Based on Fe-Montmorillonite and Its Potential Application

**DOI:** 10.3390/polym15061429

**Published:** 2023-03-14

**Authors:** Zhiming Wang, Huimin Chen, Yunpeng Zhang, Qingzhao Wang

**Affiliations:** 1 College of Chemical and Biological Engineering, Shandong University of Science and Technology, Qingdao 266590, China; 2 College of Materials Science and Engineering, Qingdao University of Science and Technology, Qingdao 266042, China

**Keywords:** Fe-MMT, PE/Fe-MMT films, photo-oxidative degradation, mechanism of molecular weight reduction, microplastics

## Abstract

The reactions occurring in the oxidative degradation phase during the photo-oxidative degradation of polyethylene (PE) are the factors leading to molecular weight reduction. However, the mechanism of molecular weight reduction before oxidative degradation has not been clarified. The present study aims to investigate the photodegradation of PE/Fe-montmorillonite (Fe-MMT) films, especially molecular weight change. The results show the rate of photo-oxidative degradation of each PE/Fe-MMT film is much faster than that of the pure linear low-density polyethylene (LLDPE) film. A decrease in the molecular weight of polyethylene was also found in the photodegradation phase. Based on this, it was found that the transfer and coupling of primary alkyl radicals originating from photoinitiation lead to a decrease in the molecular weight of polyethylene, and the kinetic results validate this new mechanism well. This new mechanism is an improvement on the existing mechanism of molecular weight reduction during the photo-oxidative degradation of PE. In addition, Fe-MMT can greatly accelerate the reduction of PE molecular weight into small oxygen-containing molecules as well as induce cracks on the surface of polyethylene films, all of which can accelerate the biodegradation process of polyethylene microplastics. The excellent photodegradation properties of PE/Fe-MMT films will be useful in the design of more environmentally friendly degradable polymers.

## 1. Introduction

Polyethylene (PE) plastic, with many desirable properties such as low cost, high strength, good barrier properties, light weight, water resistance, and high stability [1], and its disposable film products are widely used in our daily lives [2]. Polyethylene is known to be extremely difficult to degrade in the natural environment, so its use in large quantities for disposable products has caused serious environmental problems, and taking into account economic benefits and environmental issues, recycling [3,4] and incineration [5,6] are not sustainable methods. Thermal oxygen degradation is usually studied in accelerated tests at 80 °C or above [7,8], which is difficult to achieve in natural environments. In contrast, natural light can initiate the degradation of polyethylene even at room temperature. Therefore, the main degradation pathway of PE in the environment is photo-oxidative degradation.

**Scheme 1:**
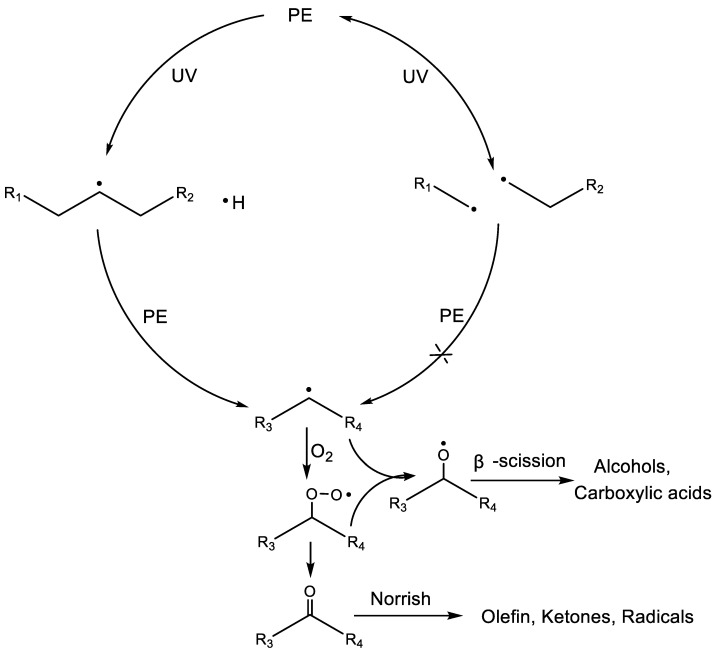
The reaction route involves the decrease of molecular weight in the process of PE photo-oxidation degradation. R1, R2, R3, and R4: long-chain alkyl.

The mechanism of photo-oxidative degradation of polyethylene was reported in detail by Costa [9] and Bracco [10]. The photo-oxidative degradation of PE begins with the photo cracking of the C-H and C-C bonds, generating H radicals, secondary alkyl macroradicals, and primary alkyl macroradicals, as shown in Scheme 1. Primary alkyl radicals regenerate C-C bonds in situ and emit heat due to site-resistance effects and mobility limitations. The reaction of secondary alkyl macroradicals with oxygen causes PE to enter the oxidative degradation stage. The series of reactions in the oxidative degradation stage generate oxygen-containing products such as ketones, aldehydes, carboxylic acids, and alcohols, which give the local chain segments of PE a polysaccharide-like structure and provide the possibility of microbial recognition. Meanwhile, the β-scission [11] of alkoxy macroradicals and the Norrish reaction of carbonyl products lead to the breakage of the polyethylene main chain, transforming the polyethylene from long-chain macromolecules to short-chain small molecules. Ideally, PE undergoes photo-oxidative degradation to form tiny fragments with polysaccharide-like structures and molecular weights below 5000 Da [12]. These tiny fragments are enriched underground and gradually recognized, digested, and eventually metabolized by microorganisms into carbon dioxide and water. However, most of the polymer fragments eventually become microplastics because they have difficulty meeting the conditions for metabolism by microorganisms before entering the ground, and polyethylene is one of the main sources of microplastics that are causing irreparable damage to freshwater lakes [13,14], groundwater [15], soil [16,17], marine ecosystems [18], and even freshwater animals [19]. Therefore, studies on the photo-oxidative degradation of PE should focus more on those properties that are favorable for biodegradation and which undoubtedly accelerate the complete degradation of PE microplastics into the water and carbon dioxide.

Indeed, the reactions occurring in the oxidative degradation phase during the photo-oxidative degradation of PE are the factors leading to molecular weight reduction. However, the mechanism of molecular weight reduction before oxidative degradation has not been clarified. Since pure polyethylene degrades very slowly under light conditions, we therefore added the photosensitizer Fe-montmorillonite (Fe-MMT) to accelerate this process. In this paper, the photo-oxidative degradation behaviors of PE/Fe-MMT films were investigated under continuous UV irradiation. A new molecular weight reduction mechanism of PE was investigated based on the photo-oxidative degradation behavior of PE/Fe-MMT films, and the potential applications of PE/Fe-MMT films are explored based on the study of the properties of degradation products. The findings from this study contribute to a better understanding of the molecular weight degradation behavior of polyethylene and provide a possible pathway for the eventual degradation of disposable PE products or microplastics to water and carbon dioxide.

## 2. Experimental Section

Materials: Linear low-density polyethylene LLDPE (SABIC, 218 W, M_v_ = 52,231 (±158)) was purchased from Shanghai Hongwei Plastics Co., Ltd., Shanghai, China. Cetyl trimethyl ammonium bromide (C_16_H_33_N^+^(CH_3_)Br(CTAB), AR), FeCl_3_·6H_2_O (AR), and ethanol (AR) were purchased from Qingdao Jingke Instrument Reagent Co., Ltd., Qingdao, China. Paraxylene (AR) was purchased from Shanghai Macklin Biochemical Co., Ltd., Shanghai, China. Sodium montmorillonite (Na-MMT) with a cation exchange capacity (CEC) of 120 mmol/100 g was purchased from Shanghai Ethyl Chemical Co., Ltd., Shanghai, China.

**Scheme 2:**
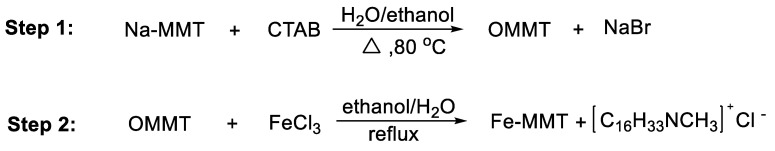
Synthesis of Fe-MMT.

Preparation of Fe-MMT: Fe-MMT was synthesized as shown in Scheme 2. Ferric montmorillonite (Fe-MMT) was prepared via two ion-exchange reactions using alkylammonium and FeCl_3_·6H_2_O. First, Na-MMT (30 g, CEC = 120 mmol/100 g) and 500 mL H_2_O were stirred in a flask to disperse at 60 °C. CTAB (16.4 g) was dissolved in anhydrous ethanol (200 mL) at 50 °C for future use. Then, the ethanol solution of CTAB was slowly added into the flask, heated to 80 °C, and refluxed for 4 h. The products were filtered and washed with 500 mL water. The filter cake was transferred to a new flask, 500 mL ethanol was added, and the solution was stirred to disperse. Then, the prepared FeCl_3_ aqueous solution (21.68 g FeCl_3_·6H_2_O dissolved in 150 mL water) was added to the system, which was raised to reflux and kept at the reaction temperature for 3 h. After the reaction was completed, the solution was filtered while it was hot and washed with 1:1 (volume ratio) ethanol/water (300 mL × 3) and ethanol (300 mL × 2), and the product was dried at 70 °C for 24 h, ground, and sifted through a 200-mesh sieve. Finally, Fe-MMT, a brick red powder (maximum diameter of 75 μm), was obtained.

**Scheme 3:**
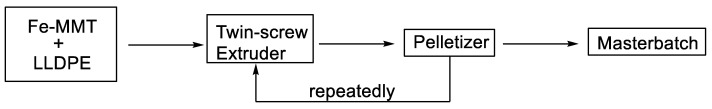
Preparation of LLDPE/Fe-MMT masterbatch.

Preparation of LLDPE/Fe-MMT (*w*/*w* = 90/10) masterbatch: The masterbatch preparation process is shown in Scheme 3. LLDPE (180.3 g) particles and Fe-MMT (20.8 g) powder mixture were first extruded using a twin-screw extruder (screw diameter = 19 mm, L/D = 40, Bau Technology, Seoul, Korea), and the residence time was about 15 min. The operating temperature of the extruder was maintained at 135 °C, 145 °C, 160 °C, 170 °C, and 180 °C from hopper to die. After cooling, the extrudates were pelletized. The formed pellets were extruded again, cooled, and granulated to obtain 180.6g of masterbatch with 10% Fe-MMT content.

**Scheme 4:**
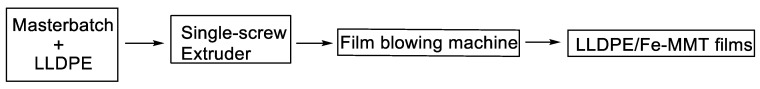
Preparation of LLDPE/Fe-MMT films.

Preparation of LLDPE/Fe-MMT films: The LLDPE/Fe-MMT films were blown in a pilot-scale film blow molding facility using a single-screw extruder with a 40 mm screw diameter rotating at 75 rpm. The L/D ratio of the screw was 25:1, and the die length was 100 mm with a 2.5 gap. The operating temperature of the extruder was controlled at C1-160 °C, C2-170 °C, and C3-180 °C, and the die temperature was set at 190 °C. The preparation process is shown in Scheme 4. The LLDPE films with different Fe-MMT contents prepared in this study are shown in Table 1. The film thickness was 12 μm.

Elongation at break test of film samples: The elongation at break (X) of PE/Fe-MMT films was tested according to ISO 20753-2018 utilizing a microcomputer control electronic universal testing machine (UTM4104) at a crosshead speed of 5 cm/min. Sample dimensions 7.5 cm length, 15 mm width, and 12 µm thickness, with 30 mm distance between two clamps. Four parallel tests were performed for each sample to take the average value.

Morphological and Chemical Characterization: The morphological structures of Na-MMT and Fe-MMT were characterized by scanning electron spectroscopy/energy dispersive spectroscopy (SEM/EDS, Apreo S HiVac, Thermo Fisher, Waltham, MA, USA). The crystal properties of Na-MMT and Fe-MMT were characterized by X-ray diffraction (XRD, D8 Advance, Bruker, Billerica, MA, USA). The chemical structures and compositions of Na-MMT and Fe-MMT were characterized by FTIR-ATR (Nicolet iS50, Thermo Fisher) and X-ray photoelectron spectroscopy (XPS, ESCALAB XI+, Thermo Fisher). Detailed characterization and analysis of Na-MMT and Fe-MMT are in the Supporting Materials.

Photo-oxidative Degradation Test: The sample films (12 μm) were used for the photo-oxidative degradation study. The UV irradiation test was carried out in a LUV-2 UV accelerated weathering tester (Shanghai Pushen Chemical Machinery Co., Ltd., Shanghai, China), which was equipped with three UV tubes (Pushen 20 W). The sample was 9 cm from the lamp, and the light intensity was 1.65 mW/cm^2^ (measured by CEL-NP2000 optical power meter). The working temperature was controlled at 40 ± 2 °C. FTIR-ATR and molecular weight measurements were taken every 24 h during 144 h of UV irradiation experiments to track the photo-oxidative degradation process of the sample films.

Infrared spectroscopy Test: The IR spectrum of the sample films were obtained using a Nicolet iS50 FT-IR spectrometer coupled with an attenuated total reflectance (ATR) accessory. For each sample, an interferogram was obtained using a ZnSe crystal with a reflection angle of 45 to maintain the same probing depth. The measurement range was from 400 to 4000 cm^−1^, with a 4 cm^−1^ resolution and a total of 32 scans.

Molecular Weight Test: The decrease in molecular weight is an important index in the degradation of polyethylene. In this study, the changes in molecular weight of the PE sample films were measured with an Ubbelohde viscometer (capillary internal meridian: 0.5–0.6 mm) during photo-oxidative degradation. Experimental conditions: Paraxylene (solvent), T = 105 °C; the concentration of PE in paraxylene is about 0.002 g/mL. The viscometric average molecular weight (M_v_) values of the samples were obtained by using the equation [20]: [η] = 0.0165 $M_{v}^{0.83}$.

## 3. Results and Discussion

### 3.1. Structure of Fe-MMT and Its Distribution in Polyethylene Matrix

**Figure 1:**
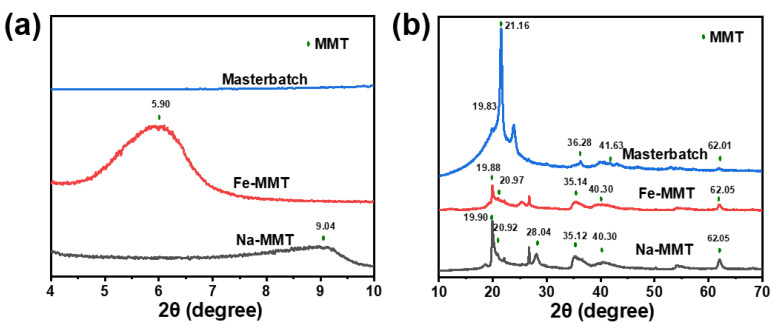
Small-angle XRD patterns (**a**) and wide-angle XRD patterns (**b**) of Na-MMT, Fe-MMT, and masterbatch.

Fe-MMT was prepared from Na-MMT by two ion exchange reactions. The power X-ray patterns of Na-MMT and Fe-MMT are shown in Figure 1. For Na-MMT, it can be well observed that the (001) diffraction peak appears at 2θ = 9.04°, corresponding to d_001_ = 0.98 nm calculated by Bragg’s Law (λ = 2dsinθ). For Fe-MMT, the characteristic peaks in the wide-angle part remain consistent with those of Na-MMT as seen in Figure 1b. However, a broad diffraction peak is located at 5.90° for the (001) plane of Fe-MMT, and that d_001_ value is 1.50 nm, which indicates that Fe-MMT possesses a larger layer spacing. In addition, as presented in Figure 1b, the XRD patterns of the masterbatch showed the disappearance of the (001) plane, suggesting that Fe-MMT was in the exfoliated state in the PE matrix. The exfoliated state allows PE molecules to be in full contact with Fe, which is beneficial to accelerate the photo-oxidative degradation process of PE.

**Figure 2:**
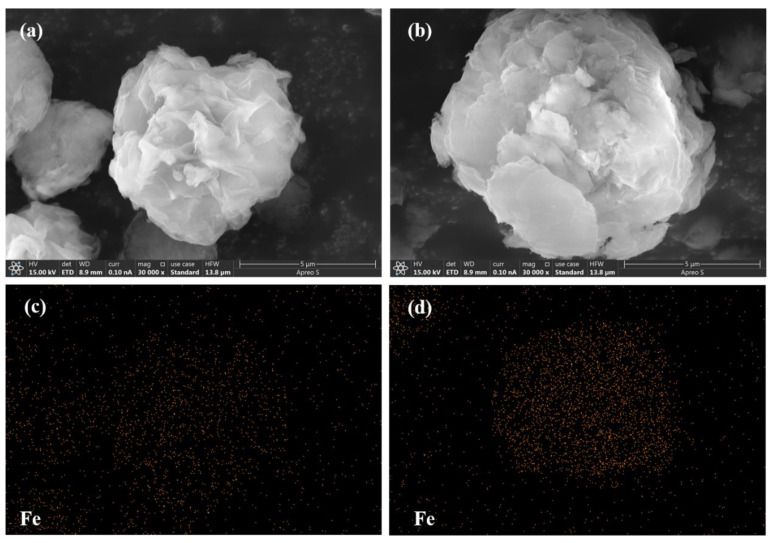
(**a**) SEM images of Na-MMT; (**b**) SEM images of Fe-MMT; (**c**) EDS images of Fe distribution in Na-MMT; (**d**) Fe distribution in Fe-MMT.

Figure 2 shows the SEM image and corresponding EDS image of the Fe distribution in Na-MMT and Fe-MMT. From the figure, it can be seen that the Fe-MMT structure contains more Fe elements and the distribution is relatively concentrated. This indicates that a large amount of Fe^3+^ was successfully introduced into the MMT structure.

**Figure 3:**
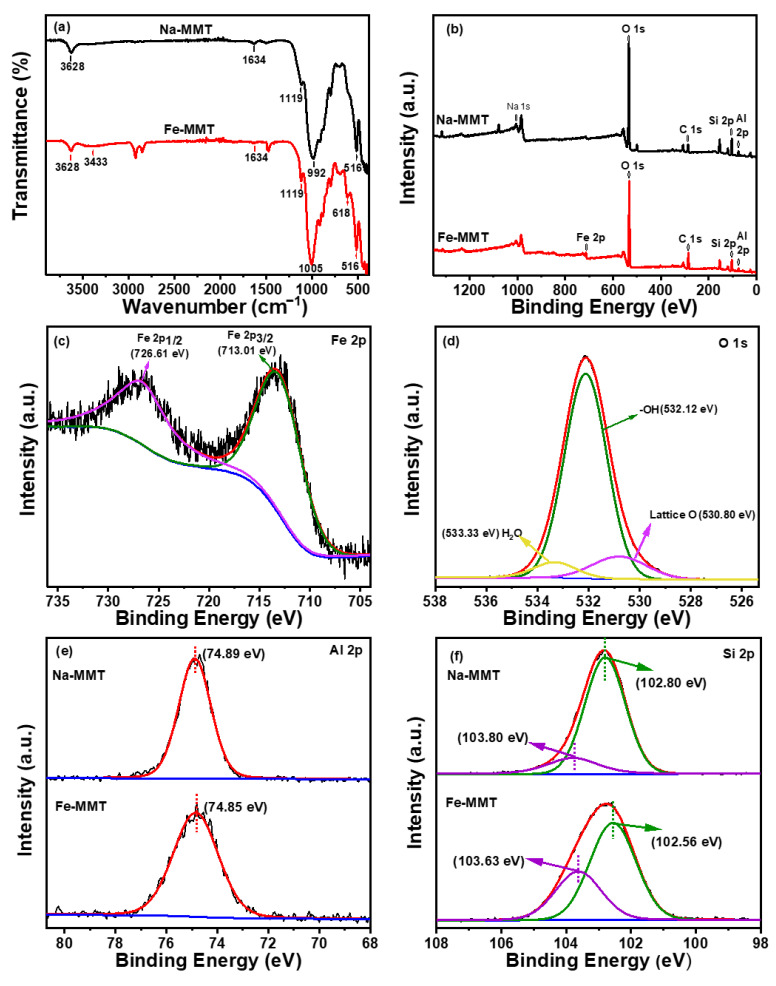
(**a**) FT-IR spectrum and (**b**) survey XPS spectrum of Na-MMT and Fe-MMT. High-resolution XPS spectrum of (**c**) Fe 2p, (**d**) O 1s for Fe-MMT, (**e**) Al 2p and (**f**) Si 2p for Na-MMT and Fe-MMT.

The chemical compositions and interface characteristics were characterized by FT-IR and XPS. The FT-IR results in Figure 3a show that the peaks at approximately 3628 cm^−1^ and 1634 cm^−1^ are associated with hydroxyl groups. The band peak of 3433 cm^−1^ for Fe-MMT is related to physically or chemically adsorbed water. In addition, the stretching vibration band of O-Si-O at 992 cm^−1^ and the vibration band of Si-O-Si at 1119 cm^−1^ indicate the layered structure of silicon-oxygen tetrahedron in the montmorillonite. The peak at 516 cm^−1^ attributed to Si-O-Al is also detected. The presence of a new peak at 618 cm^−1^ in the spectrum of Fe-MMT is related to the stretching vibration of the Fe-O bond [21].

The surface chemical element compositions and chemical states of Na-MMT and Fe-MMT were characterized by XPS. Survey scan results, as shown in Figure 3b, showed that Fe-MMT not only has Al, Si, and O elements representing the MMT phase but also has obvious characteristic peaks of Fe elements compared to Na-MMT. The Fe 2p core-level spectra revealed that the iron atoms were in the formal chemical valance state of +3 in the interlayer of montmorillonite, and the binding energies of Fe 2p3/2 and Fe 2p1/2 were located at 713.01 and 726.61 eV (Figure 3c), which corresponded to the core-level spectra of Fe^3+^ ions in their oxide or hydroxide form. The high-resolution O 1s spectrum of Fe-MMT (Figure 3d) also confirmed the existence of O atoms in two chemical states of lattice oxygen (O^2−^) and hydroxyl (-OH) with binding energies of 530.80 eV and 532.12 eV, respectively [22]. The binding energy of 533.33 eV was attributed to chemically absorbed water or ligand water (H_2_O-Fe) [23]. Figure 3e,f show the high-resolution spectra of Al 2p and Si 2p for Na-MMT and Fe-MMT. It can be seen that the binding energy of Al 2p exhibits no obvious change from Na-MMT to Fe-MMT, while the binding energy of Si 2p exhibits a slight shift. This result indicates that Si-O-Fe bonds are formed in the layers of montmorillonite. The binding energies corresponding to all relevant XPS peaks are shown in Table 2.

**Figure 4:**
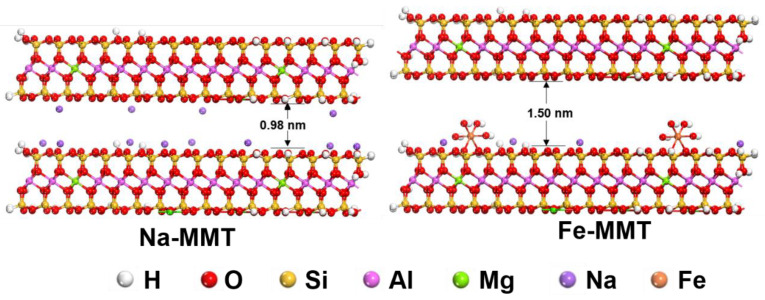
Structure of Na-MMT and a possible structure of Fe-MMT.

**Figure 5:**
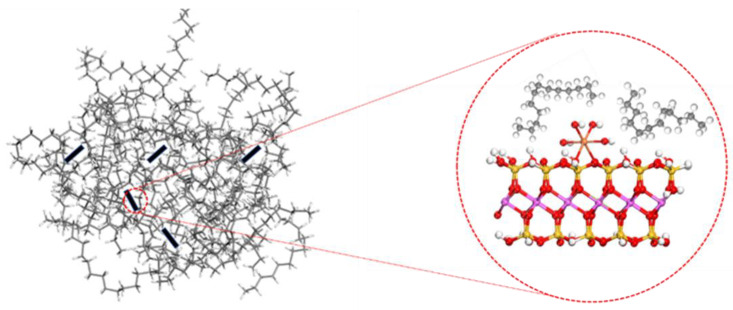
Fe-MMT distribution in masterbatch or film samples.

Based on the above analysis, we derived a possible structure of Fe-MMT, as shown in Figure 4. In addition, the XRD results of the masterbatch showed the disappearance of (001) crystalline surface characteristic peaks, which indicated that Fe-MMT was in the exfoliated state (as shown in Figure 5) in the PE matrix. The exfoliated state allows PE molecules to be in full contact with Fe, which is beneficial to accelerate the photo-oxidative degradation process of PE.

In addition, the test results in Table 3 show that the addition of Fe-MMT does not affect the tensile properties of polyethylene, indicating that the practicality of PE/Fe-MMT films is not limited.

### 3.2. Analysis of Irradiation Test Results

**Figure 6:**
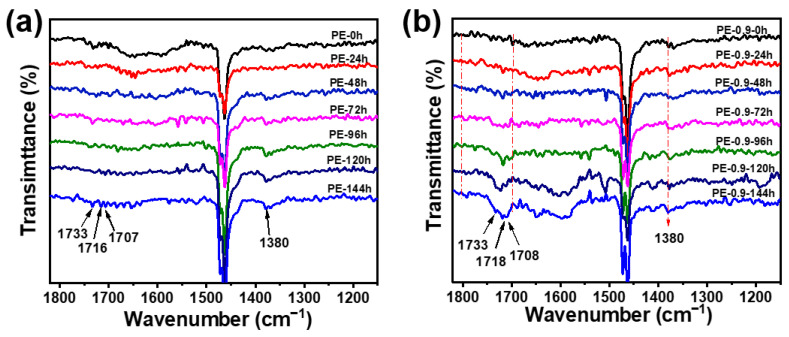
FT-IR spectra under different UV irradiation times. (**a**) LLDPE film; (**b**) PE-0.9 film.

PE sample films containing Fe-MMT at concentrations of 0, 0.1, 0.3, 0.5, 0.7, and 0.9% were used in a 144 h UV irradiation experiment and analyzed by FTIR-ATR. Figure 6a,b show the IR spectrum (1820–1150 cm^−1^) of pure LLDPE film and PE-0.9 film during UV irradiation for 144 h (other IR spectra are shown in Figures S2, S3, S4 and S5, respectively), and the wavenumber labeled in Figure 6 and their corresponding attributions are listed in Table 4. For the pure LLDPE film, weak vibration peaks belonging to carbonyl compounds are not detected until the end of the UV irradiation experiment. This means that the photoreaction process is in progress. In this process, H radicals and alkyl macroradicals are formed by the cleavage of the C-H bond or C-C bond that participates in the subsequent oxidation reaction. With the progress of the experiment, a large number of secondary alkyl macroradicals accumulated in the PE matrix and reacted with oxygen to generate alkyl peroxy macroradicals, and PE entered the auto-oxidative degradation stage. As shown in Figure 6a, the vibration peaks of oxygen-containing products were detected in the infrared spectrum after the experiment was carried out for 120 h. This indicates that pure LLDPE still needs to undergo quite a long time of photodegradation to accumulate enough secondary alkyl macroradicals to enter the oxidative degradation stage even under ultraviolet irradiation.

To gain a better overall understanding of PE photo-oxidative degradation, we investigated the degradation behavior of PE sample films with different Fe-MMT contents under the same aging conditions. For the sample of PE-0.9 film, in the initial stage of the photo-oxidative degradation experiment, the peak in the carbonyl-compound vibration peak region (red dotted line area) is almost negligible. An obvious new peak appears at 1718 cm^−1^ after 24 h UV irradiation that belongs to the C=O stretching vibration of a ketone group [24] and grows in intensity with extended UV irradiation, which indicates that the oxidative degradation process is underway. With the extension of irradiation time, other new peaks appear at 1733 cm^−1^ when the irradiation time is 48 h and at 1708 cm^−1^ and 1780 cm^−1^ when the irradiation time is 72 h. This implies that new carbonyl compounds are formed with the degradation of PE. From the early relevant works of the literature, we can learn that the peak at 1733 cm^−1^ is assigned to C=O stretching vibrations in aldehydes or esters; the peaks at 1708 cm^−1^ and 1780 cm^−1^ belong to carboxylic acid groups and γ-lactones, respectively [25,26]. The intensities of these peaks increase with increasing reaction time. In summary, the oxidation reaction introduces various oxygen-containing functional groups into the polyethylene molecular chain, making the polyethylene molecule polar and much more hydrophilic.

The infrared spectra can respond well to the changes of functional groups during the oxidative degradation of PE. For the photo-oxidative degradation of PE, the earlier the oxygen-containing functional groups are detected (indicating that the earlier the oxidative degradation of the sample starts), the faster the overall degradation rate is. The time at which the oxygen-containing functional groups were detected during the photo-oxidative degradation of different samples is shown in Table 5. From the table, we can see that the addition of Fe-MMT shortens the time for PE to enter oxidative degradation and accelerates its overall degradation rate.

### 3.3. Analysis of Molecular Weight Changes

**Figure 7:**
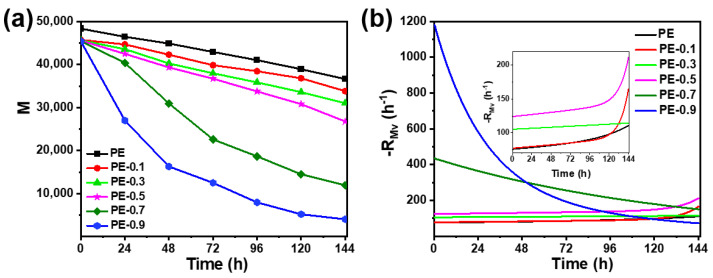
(**a**) Molecular weight changes of LLDPE film and PE-Fe-MMT sample films under different UV irradiation times and (**b**) the corresponding rate curves.

The decrease in molecular weight due to the scission of the molecular backbone is an important signal of polymer degradation. The molecular weight variation curves of different samples and the corresponding rate curves are shown in Figure 7a,b, respectively, and the specific M_v_ data are listed in Table 6. Combined with Table 6, we also found that the molecular weights of the PE/Fe-MMT samples decreased by approximately 6% compared to this of the PE sample before the UV irradiation experiment. This is due to the mechanical force or thermal degradation effect [27] between the Fe-MMT inorganic particles and LLDPE during melting, resulting in slight decreases in the molecular weights of the PE/Fe-MMT films.

From Figure 7a, it can be seen that the change of molecular weight for pure LLDPE samples was small throughout the photo-oxidative degradation experiments. The addition of Fe-MMT can effectively accelerate the reduction of PE molecular weight, but the acceleration effect is related to the amount of Fe-MMT added. It is obvious from Figure 7b that the rate of PE molecular weight reduction was greatly improved when the addition amount of Fe-MMT was greater than 0.5%. Moreover, the molecular weight reduction trend as well as the rate of low Fe-MMT content sample films have similar results with pure LLDPE samples, but all of them show better photo-oxidative degradation effects than pure LLDPE samples. Overall, after 144 h UV aging experiments, the molecular weight of pure LLDPE samples decreased by 24%, while the molecular weight of samples with 0.9% Fe-MMT addition decreased by 91%. All of the above show that Fe-MMT also plays a pivotal role in reducing the molecular weight of PE. 

It is noteworthy that the molecular weight changes of all samples were continuously decreasing, even before the oxidative degradation phase. This phenomenon is more easily observed for pure LLDPE samples, where the molecular weight decreases by about 20% before the oxidative degradation phase. Similar results were obtained for the samples with low Fe-MMT content (0.1%, 0.3%, and 0.5%). As shown in Figure 7b, it is clear that both pure PE samples and samples with low Fe-MMT content maintain a certain rate of molecular weight reduction before oxidative degradation. In addition, the molecular weight reduction rate increased significantly after entering the oxidative degradation stage, which is due to the β-scission of alkoxyl giant radicals and the Norrish reaction of carbonyl products in the oxidation stage, which can lead to the breakage of the PE backbone. These results suggest that there are also reactions that can lead to effective backbone breakage of PE molecules in the photodegradation stage. For samples with high Fe-MMT content, oxidative degradation can occur quickly due to the action of Fe-MMT, and its oxidative degradation is almost simultaneous with photodegradation. Therefore, it possesses a very high rate of molecular weight reduction at the early stage of photo-oxidative degradation. So, the study of the mechanism of molecular weight reduction during the photo-oxidative degradation of polyethylene cannot be limited to the reaction in the oxidation stage. The reactions leading to the molecular weight reduction of polyethylene are also present in a series of photochemical reactions during photodegradation.

### 3.4. Mechanism of Decreasing PE Molecular Weight under Continuous UV Irradiation

**Scheme 5:**
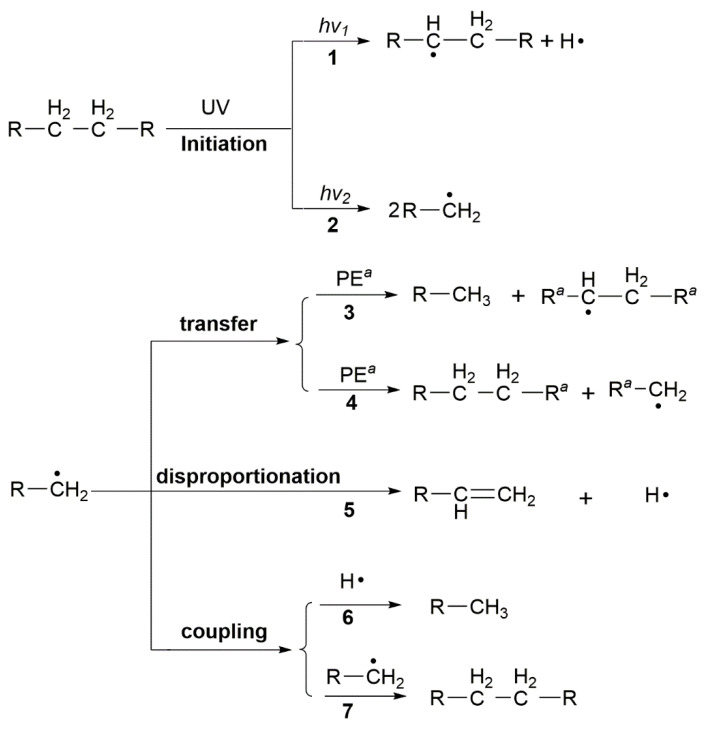
Reaction route involving the decrease in molecular weight in the process of PE photo-oxidation degradation. R, R^a^ Long-chain alkyl; ^a^ adjacent.

Bond dissociation energies for C-C bonds (375 kJ/mol) and C-H bonds (420 kJ/mol) correspond to UV radiation at 320 nm and 290 nm [28]. The photochemical reactions during the photodegradation period of LLDPE with a UV light source start from the homolytic cleavage of C-H or C-H bonds, as in reactions **1** and **2,** shown in Scheme 5. During the photodegradation period, the transfer, disproportionation, and coupling processes involved in secondary alkyl macroradicals do not result in the scission of the PE backbone. Therefore, investigating the fate of the primary alkyl macroradicals will help us to understand the mechanism of PE molecular weight reduction before the oxidative degradation period. The transfer to the adjacent carbon chain by an “H-extraction” process is accompanied by the formation of a secondary alkyl macroradical and a methyl end group (Scheme 5, reaction **3**). Another possible transfer method is to react with adjacent carbon chains to generate new primary alkyl macroradicals, which is insensitive to changes in PE molecular weight (Scheme 5, reaction **4**). Due to their extremely short lifetime, primary alkyl macroradicals are more prone to rapid decay through coupling or disproportionation. Termination by coupling of two macroradicals with the reformation of a C-C bond is widely recognized (Scheme 5, reaction **7**) [9], and coupling of primary alkyl macroradicals and H radicals with the formation of a new methyl end group is also a reasonable way (Scheme 5, reaction **6**) because of its very small size (less than 1 angstrom), high activity, and high mobility of H radicals, which is a notable efficient pathway for PE molecular weight reduction. Disproportionation (Scheme 5, reaction **5**) is only theoretically possible because no relevant disproportionation product formation was detected during the oxidation induction period. Therefore, reaction **2**, reaction **3,** and reaction **6** in Scheme 5, which originate from photoinitiated reactions, may be another new reaction mechanism for polyethylene molecular weight reduction. Based on this, through the analysis of reaction products, combined with constant light source conditions, there should be a positive correlation between the decreasing rate of polyethylene molecular weight and the increasing rate of the methyl group. To verify this mechanism, kinetic analysis of molecular weight and methyl content changes is essential.

**Figure 8:**
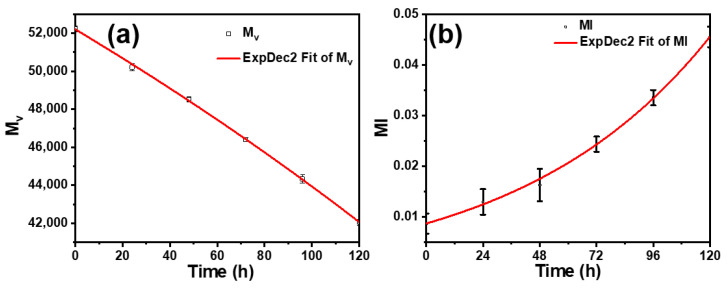
(**a**) Molecular weight change curves and (**b**) methyl index (MI) change curve of PE film. Scatter: experimental data. Red line: fitting by ExpDec2 model.

To better study the relationship between methyl end groups and molecular weight changes before the oxidative degradation period, a pure LLDPE film sample with a longer photoreaction period (about 120 h, as shown in Figure 6a) was used as the research object. The methyl index (MI) is defined as the absorbance ratio of the methyl (approximately 1380 cm^−1^, as shown in Figure 6) and methylene groups (1462 cm^−1^) according to the equation MI = A1380 cm^−1^/A1435 cm^−1^. MI calculation of LLDPE film concerns the baseline method for calculating the carbonyl index [29]. Figure 8b shows the variation of methyl content of PE sample film with UV irradiation time. It can be seen from the figure that the methyl content increases steadily with the increase of UV irradiation time and is accompanied by the decrease of polyethylene molecular weight. 

**Figure 9:**
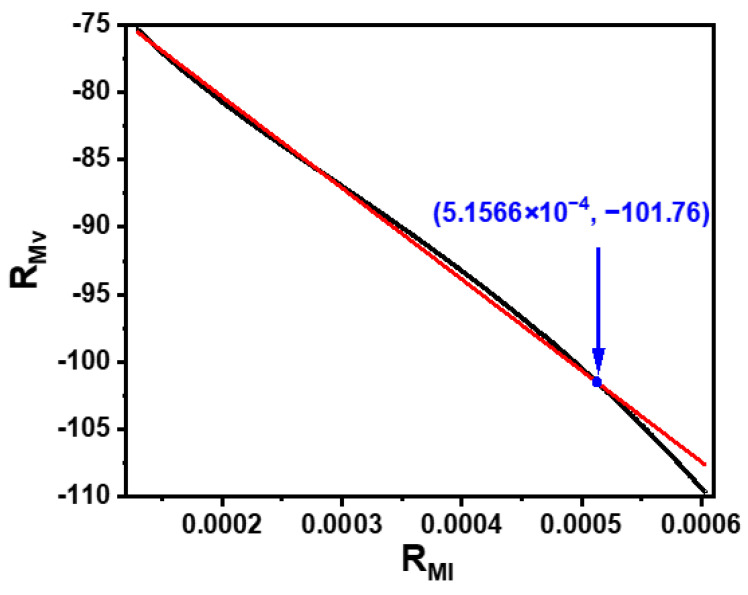
Relationship between R_Mv_ and R_MI_ of PE sample films in the photoreaction stage. Black: scatter. Red: fitting curve, adj. R-square = 0.9972.

The molecular weight reduction rate R_Mv_ = dMv/dt and the methyl index change rate R_MI_ = dMI/dt were obtained by differentiating the fitted curve, and the relationship between R_Mv_ and R_MI_ is shown in Figure 9. It can be seen from the figure that RMI is a function of RMv, and the linear fit of the scatter plot shows that they have a good linear relationship, as shown in Equation (1). In addition, after the intersection (blue point) of the two curves in the figure, the molecular weight reduction rate increases, and the fitting effect deteriorates significantly. This indicates that, in addition to the ongoing reactions 3 and 6 in Scheme 5, new reactions leading to the reduction of PE molecular weight occur after the time (about 108 h) corresponding to the intersection point. These newly occurring reactions could be the β-scission of alkoxy macroradicals [9,11] or the Norrish reaction of carbonyl products [10]. The above validates the correctness of our proposed new reaction mechanism for PE molecular weight reduction. This mechanism is complementary to the existing molecular weight reduction mechanism in the photo-oxidative degradation of PE.

(1) $$R_{\mathrm{Mv}}=-{67,649R}_{\mathrm{MI}}-67$$

**Figure 10:**
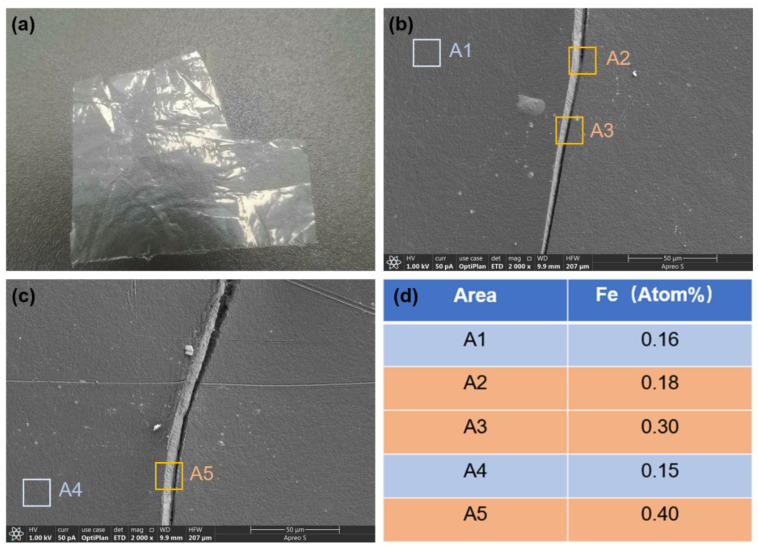
(**a**) Picture of PE-0.9 film after 72 h UV irradiation; (**b**,**c**) pictures of cracks; (**d**) the relative atomic content of Fe element.

In addition, the accelerating effect of Fe-MMT addition on the molecular weight reduction of polyethylene is tremendous, especially at high addition levels. The rapid reduction of molecular weight implies a large number of breaks in the polyethylene backbone, which can lead to a large number of cracks on the polyethylene film surface. Scanning analysis of the PE-0.9-72 h sample film surface verified the crack generation, and the results are shown in Figure 10b,c. Additionally, a total of five random regions were taken at the cracked and non-cracked areas for the energy dispersive spectroscopy (EDS) analysis of C, O, and Fe elements. The relative atomic content of Fe elements in each region is shown in Figure 10d. The results show that the elemental content of Fe in the cracked area is significantly higher than that in the uncracked area, which indicates that Fe-MMT can induce a large number of cracks in the degradation process of polyethylene film, and the generation of cracks inevitably accelerates the rate of oxygen entering the polyethylene matrix, thus accelerating the rate of oxidative degradation of polyethylene. In summary, Fe-MMT can greatly accelerate the reduction of PE molecular weight into small oxygen-containing molecules as well as induce cracks on the surface of polyethylene films, all of which can accelerate the biodegradation process of polyethylene microplastics [30]. Therefore, Fe-MMT not only has a good accelerating effect on the photo-oxidative degradation of PE but also can make the degraded PE microplastics possess more favorable properties for microbial degradation. So, the addition of Fe-MMT to PE not only greatly improves its photo-oxidative degradation rate but also has a positive effect on reducing the accumulation of polyethylene microplastics in the ground.

## 4. Conclusions

In our study, Fe-MMT was prepared by a cation exchange reaction, and its morphology and structure were characterized by FT-IR, SEM, XRD, and XPS. Moreover, Fe-MMT is in the exfoliated state in the polyethylene matrix, so Fe can be in full contact with polyethylene molecules, which is more conducive to the photo-oxidative degradation of PE. The rate of photo-oxidative degradation of each PE/Fe-MMT film is much faster than that of the pure LLDPE film. The transfer and coupling of primary alkyl radicals originating from photoinitiation lead to a decrease in the molecular weight of polyethylene, and the kinetic results validate this mechanism well. This new mechanism is an improvement on the existing mechanism of molecular weight reduction during the photo-oxidative degradation of PE. In addition, Fe-MMT can greatly accelerate the reduction of PE molecular weight into small oxygen-containing molecules as well as induce cracks on the surface of polyethylene films, all of which can accelerate the biodegradation process of polyethylene microplastics. It is believed that these properties of Fe-MMT are important for the study of the biodegradation of polyolefin MPs. The excellent photodegradation properties of PE/Fe-MMT films will be useful in the design of more environmentally friendly degradable polymers.

## Figures and Tables

**Table 1:** Compositions of the sample films used in this work.

Sample Name	LLDPE (wt%)	Masterbatch (wt%)	Fe-MMT (wt%)
PE	100	0	0
PE-0.1	99	1	0.1
PE-0.3	97	3	0.3
PE-0.5	95	5	0.5
PE-0.7	93	7	0.7
PE-0.9	91	9	0.9

**Table 2:** The binding energies corresponding to some of the peaks in the XPS spectra.

Sample	Binding Energy (ev)			
	Fe 2p	O 1s	Si 2p	Al 2p
Na-MMT	--	--	103.80 102.80	74.89
Fe-MMT	726.61 (2p1/2) 713.01 (2p3/2)	533.33 (H_2_O) 532.12 (-OH) 530.80 (Lattice O)	103.63 102.56	74.85

**Table 3:** Initial elongation at break of PE/Fe-MMT films.

Sample	PE	PE-0.1	PE-0.3	PE-0.5	PE-0.7	PE-0.9
X (%)	328 ± 26	319 ± 30	318 ± 6	306 ± 6	327 ± 12	342 ± 15

**Table 4:** Vibrational peaks in infrared spectra and their attribution.

Wavenumber (cm^−1^)	Attribution
1780	γ-lactones
1733	aldehydes or esters
1716	ketones
1707	carboxylic acids
1380	methyl

**Table 5:** The time of infrared detection of carbonyl functional groups in different samples.

Sample	Time (h) ^a^
PE	72
PE-0.1	72
PE-0.3	48
PE-0.5	48
PE-0.7	24
PE-0.9	24

^a^ The actual time is much less, especially for PE-0.9 samples.

**Table 6:** Mv Changes of PE and PE/Fe-MMT films under different UV irradiation times.

Sample	Mv at UV Irradiation Time						
	0 h	24 h	48 h	72 h	96 h	120 h	144 h
PE	52,231	50,212	48,517	46,401	44,334	42,066	39,584
PE-0.1	49,455	48,324	45,704	43,076	41,562	39,912	36,568
PE-0.3	49,329	47,102	43,514	41,035	38,755	36,301	33,515
PE-0.5	49,237	46,027	42,506	39,689	36,469	33,294	28,933
PE-0.7	49,150	43,642	33,455	24,413	20,160	15,660	12,888
PE-0.9	49,090	29,159	17,600	13,511	8587	5600	4344

## Data Availability

The data presented in this study are available on request from the corresponding author.

## Supplementary Materials

The following supporting information can be downloaded at: https://www.mdpi.com/article/10.3390/polym15061429/s1, Figure S1. Changes of carbonyl infrared vibration peaks of different samples after UV irradiation for 96 h; Figure S2. FT-IR spectra of PE-0.1 film under different UV irradiation times; Figure S3. FT-IR spectra of PE-0.3 film under different UV irradiation times; Figure S4. FT-IR spectra of PE-0.5 film under different UV irradiation times; Figure S5. FT-IR spectra of PE-0.7 film under different UV irradiation times.
