# Supplementary material for: Study on the Mechanism of Molecular Weight Reduction of Polyethylene Based on Fe-Montmorillonite and Its Potential Application
Source: Polymers (Basel). 2023 Mar 14;15(6):1429. doi: 10.3390/polym15061429 (PMC10057148; doi:10.3390/polym15061429)
Supplement: Supplementary file 1 [file polymers-15-01429-s001.zip › polymers-2273079-supplementary.pdf]

Supporting Information

Study on the Mechanism of Molecular Weight Reduction of Polyethylene Based on Fe-Montmorillonite and Its Potential Application

Zhiming Wang [1], Huimin Chen [2], Yunpeng Zhang [1] and Qingzhao Wang [1,*]

[1] College of Chemical and Biological Engineering, Shandong University of Science and Technology, Qingdao 266590, China; wang_zhiming@126.com (Z.W.); zhang1961485@126.com (Y.Z.)

[2] College of Materials Science and Engineering, Qingdao University of Science and Technology, Qingdao 266042, China; chen_huiminmm@163.com

* Correspondence: qzhwang@sdust.edu.cn

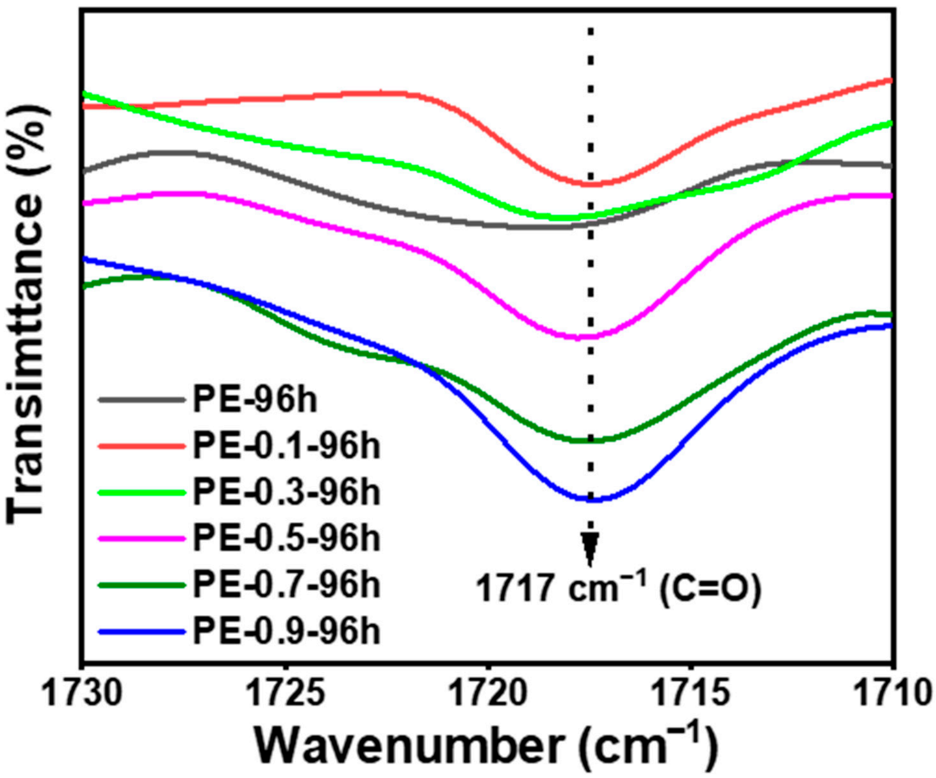

Figure S1. Changes of carbonyl infrared vibration peaks of different samples after UV irradiation for 96 h.

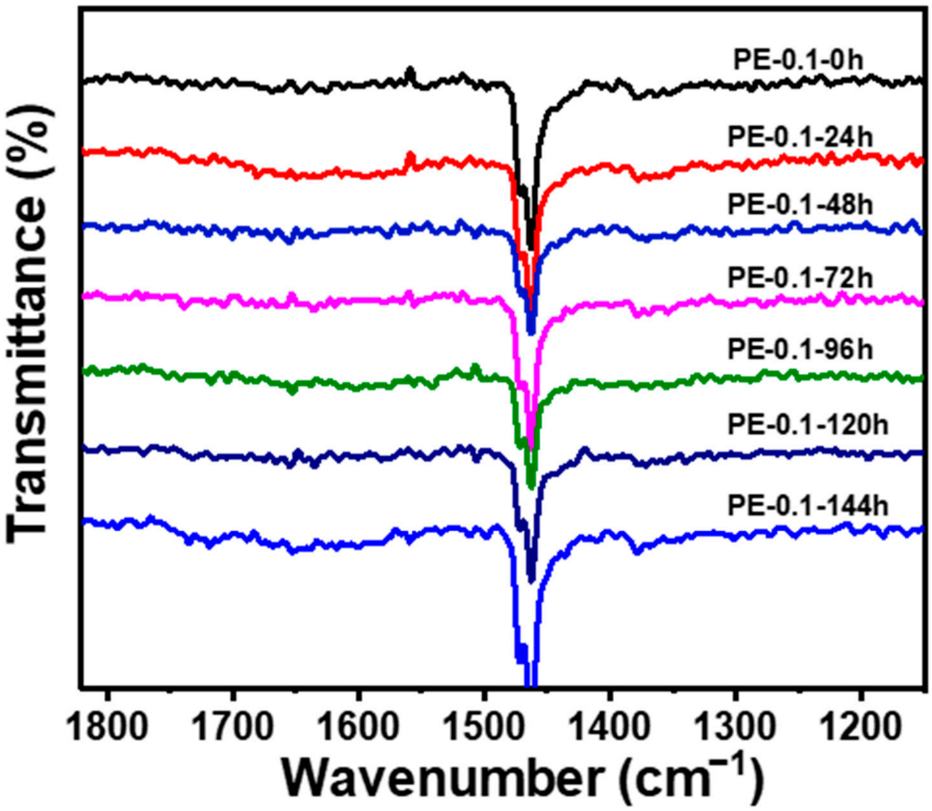

Figure S2. FT-IR spectra of PE-0.1 film under different UV irradiation times.

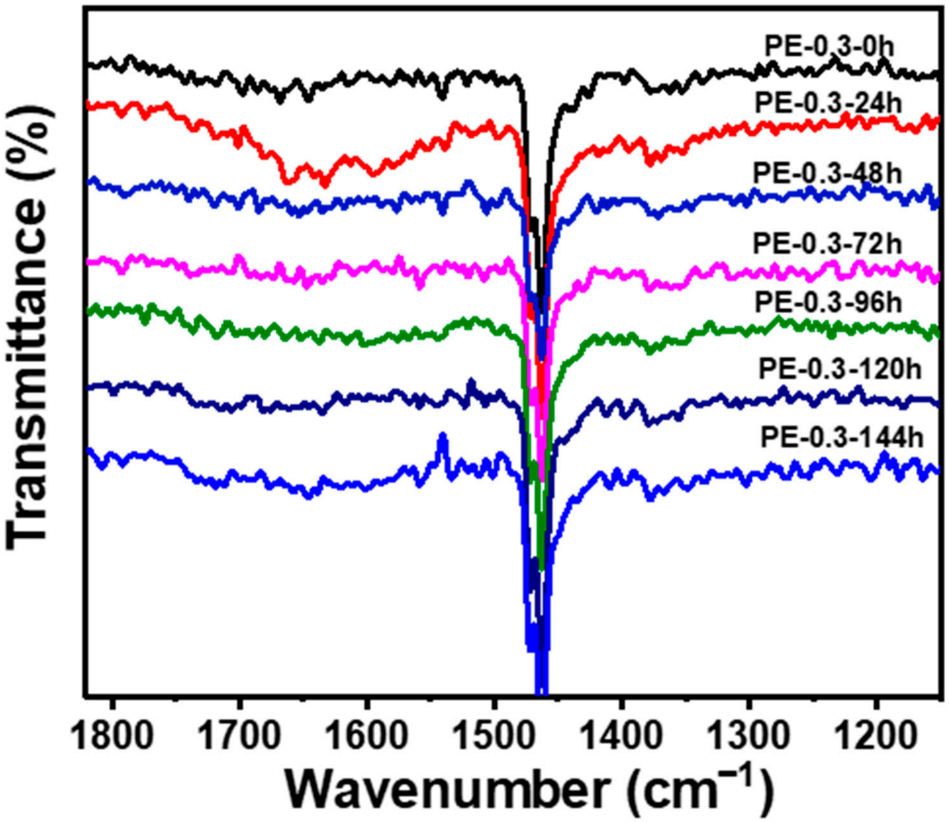

Figure S3. FT-IR spectra of PE-0.3 film under different UV irradiation times.

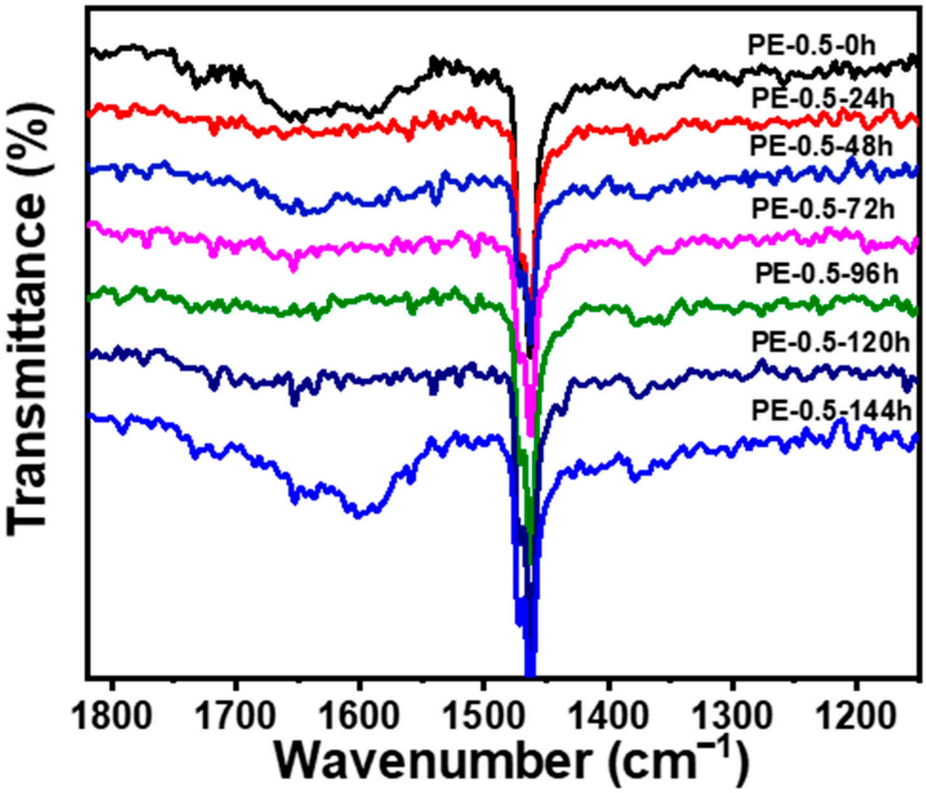

Figure S4. FT-IR spectra of PE-0.5 film under different UV irradiation times.

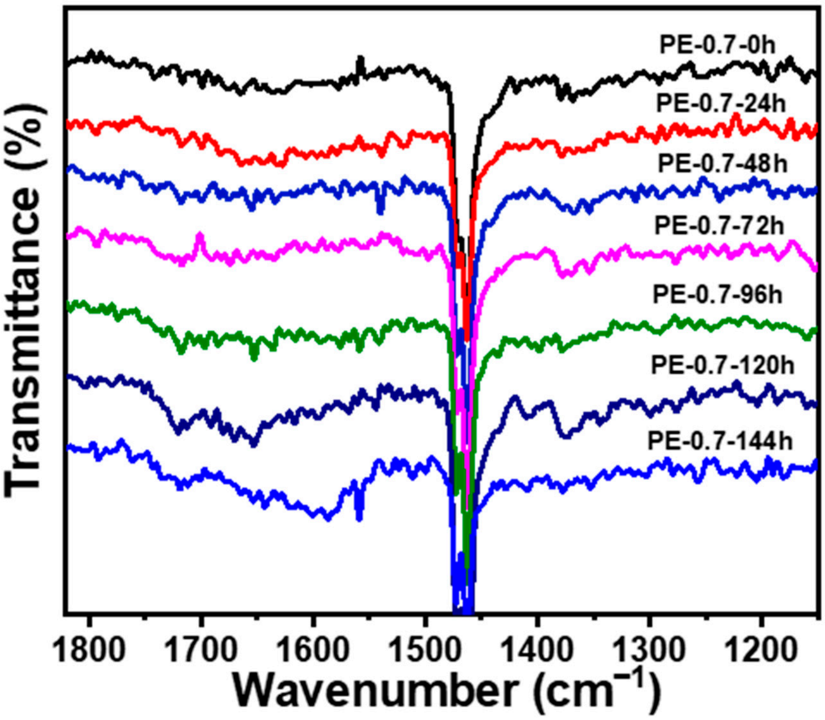

Figure S5. FT-IR spectra of PE-0.7 film under different UV irradiation times.
